# Assessment of efficacy and safety by CalliSpheres versus HepaSpheres for drug-eluting bead transarterial chemoembolization in unresectable large hepatocellular carcinoma patients

**DOI:** 10.1080/10717544.2021.1943057

**Published:** 2021-06-28

**Authors:** Guangsheng Zhao, Song Liu, Songbai Chen, Zhizhong Ren, Chuang Li, Jie Bian, Jianlin Wu, Jun Zhou, Yuewei Zhang

**Affiliations:** a Cancer Interventional Center, Affiliated Zhongshan Hospital of Dalian University, Dalian, China; b Cancer Interventional Center, Linyi Cancer Hospital, Linyi, China; c Hepatobiliary and Pancreatic Center, Beijing Tsinghua Changgung Hospital, Beijing, China; d Department of Radiology, The Second Affiliated Hospital of Dalian Medical University, Dalian, China; e Department of Radiology, Affiliated Zhongshan Hospital of Dalian University, Dalian, China

**Keywords:** Large hepatocellular carcinoma, drug-eluting bead transarterial chemoembolization, CalliSpheres, HepaSpheres, efficacy and safety

## Abstract

This study aimed to compare efficacy and safety of HepaSpheres and CalliSpheres in unresectable large hepatocellular carcinoma (HCC) patients. One hundred and twenty-seven unresectable large HCC patients receiving drug-eluting bead transarterial chemoembolization (DEB-TACE) treatment with CalliSpheres or HepaSpheres microspheres were analyzed. Treatment response, Karnofsky performance status (KPS) score, adverse events, main liver function indexes, time to progression (TTP), and overall survival (OS) were analyzed. Objective response rate (82.7% vs. 63.8%, *p*=.030) and disease control rate (100.0% vs. 91.5%, *p*=.030) were increased in CalliSpheres group compared to HepaSpheres group at 1 month after treatment, while no difference was found between the two groups regarding treatment response at 3 or 6 months post treatment (all *p*>.05). The KPS score at 1, 3, and 6 months was similar between the two groups (all *p*>.05). As for the liver function, the ALT, AST, ALB, and TBIL levels at 7 and 30 days were of no difference between the two groups (all *p*>.05). In addition, the adverse events including nausea/vomiting, pain, fever, myelosuppression, biloma, and abscess were of no difference between the two groups, either (all *p*>.05). In terms of survival profile, there was no difference regarding TTP (6.3 months (95%CI: 5.9–6.6 months) vs. 6.0 months (95%CI: 5.6–6.4 months), *p*=.082) or OS (23.0 months (95%CI: 20.1–25.9 months) vs. 22.0 months (95%CI: 20.2–23.8 months), *p*=.571) between the two groups. In conclusion, CalliSpheres seems to be superior in short-term efficacy and equal in long-term efficacy as well as safety compared to HepaSpheres for DEB-TACE treatment in unresectable large HCC patients.

## Introduction

Nowadays, liver cancer is one of the world’s most frequent and lethal cancer with an estimated incidence of 9.3 and a mortality of 8.5 per 100,000 person-years, and hepatocellular carcinoma (HCC) is the predominant type of liver cancers (Bray et al., 2018). HCC treatment is predominantly based on the Barcelona Clinic Liver Cancer (BCLC) staging system, and patients in the intermediate-late stage are the most common in the clinical setting; however, the treatment options for these patients are quite limited (Pinero et al., 2018). In recent decades, the introduction of transarterial chemoembolization (TACE) has vastly improved the HCC patients’ prognosis, especially for intermediate-late stage patients, among which, despite that conventional TACE is the most common in the clinical setting, drug-eluting bead TACE (DEB-TACE) is recently becoming more and more popular thanks to its benefit in treatment responses and tolerance profile (Li et al., 2019; Xiang et al., 2019; Wang et al., 2020). Nonetheless, whether the category of microspheres used in DEB-TACE has impact on efficacy and safety in HCC patients is largely unknown.

HepaSpheres and CalliSpheres are two competitive microsphere products in the treatment using DEB-TACE among HCC patients. HepaSpheres has been applied in the clinical practice for almost 30 years, it is a vinyl alcohol-sodium acrylate microsphere featured by good quality in chemotherapeutic absorbing/releasing abilities and its acceptable flexibility in the vessels (Jordan et al., 2010; Zurstrassen et al., 2017). As for CalliSpheres, it is the first microsphere product completely produced in China and utilized for DEB-TACE in recent years, and there are many clinical studies and trials elucidating that it presents with satisfying efficacy and safety in DEB-TACE treatments for HCC patients (Ren et al., 2019; Zhang et al., 2019; Duan et al., 2020; Liang et al., 2020). Moreover, the DEB-TACE using microsphere products also presents with considerable efficacy and tolerance in some advanced HCC patients, such as the patients with large HCC tumor (Song et al., 2011). However, to our best knowledge, the comparison of efficacy or tolerance between HepaSpheres and CalliSpheres in large HCC patients has not been done.

Therefore, the aim of the present study was to compare the efficacy and safety of HepaSpheres and CalliSpheres in unresectable large HCC patients.

## Materials and methods

### Study population

In this multicenter retrospective study, we analyzed 127 unresectable large HCC patients who received DEB-TACE treatment with CalliSpheres or HepaSpheres microspheres in Affiliated Zhongshan Hospital of Dalian University, Beijing Tsinghua Changgung Hospital, and Linyi Cancer Hospital, from July 2016 to July 2018. Inclusion criteria were as follows: (a) diagnosed as primary HCC; (b) BCLC stage B or C; (c) maximum diameter of a single tumor ≥5 cm; (d) Child-Pugh stage A or B; (e) age ≤80 years; (f) Eastern Cooperative Oncology Group performance status (ECOG PS) score of 0–2 points; (g) treated by DEB-TACE with CalliSpheres or HepaSpheres microspheres. Exclusion criteria included (a) previously underwent radiofrequency ablation or radio-chemotherapy before TACE; (b) complicated with other malignancies; (c) data missing including clinical data, imaging evaluations, or follow-up records. Among 127 patients, 67 patients who received DEB-TACE with CalliSpheres were categorized as CalliSpheres group, and 60 patients who received DEB-TACE with HepaSpheres were categorized as HepaSpheres group. The study complied with the Declaration of Helsinki, and the Ethics Committee of the Affiliated Zhongshan Hospital of Dalian University (principal research center) approved it. All patients provided the written informed consent forms.

### Clinical data collection

Medical records of patients were reviewed to collect the basic clinical features for study analysis, which mainly included age, gender, history of liver disease, ECOG PS score, model for end-stage liver disease (MELD) score, Child-Pugh stage, tumor size, numbers of tumors, vascular invasion status, extrahepatic metastasis status, BCLC stage, and preoperative alpha-fetoprotein (AFP).

### DEB-TACE treatment

Before operation, drug-loading was performed. For CalliSpheres group, 50 mg pirarubicin solution was mixed with 1.0 g CalliSpheres (diameter: 300–500 μm, Suzhou Hengrui Galisheng Biomedical Technology Co., Ltd., Suzhou, China), then the contrast agent was added in a ratio of 1:1, and the mixture was placed for 30 minutes for further use. For HepaSpheres group, ‘4-fold method’ was used for drug-loading, as follows: 50 mg pirarubicin was dissolved in 20 mL of 0.9% NaCl, then 10 mL of the solution was injected into a bottle of HepaSpheres (1.0 g, diameter: 50–100 μm, Biosphere Medical, Inc., South Jordan, UT), followed by shaking up every three minutes for 10 minutes. The remaining 10 mL solution of pirarubicin was extracted into a syringe, next, the suspension in the bottle was also extracted into the syringe, followed by shaking up every five minutes for 15 minutes. After that, the mixture in the syringe was transferred to operating table, followed by full sedimentation. Subsequently, the liquid supernatant was pushed out, and the remaining drug-loaded HepaSphere was diluted with 20 mL contrast agent for further use. After preparation of drug-eluting microspheres, DEB-TACE was carried out. In brief, arterial angiography was performed to identify all arteries that supply blood to the tumor, then the microcatheter was introduced into the target vessel by superselective catheterization. Following that, the prepared drug-eluting microspheres and contrast agent were injected into the target vessel slowly and alternately until the complete disappearance of tumor staining. When necessary, gelatin sponge particles were used for supplementary embolization. Criteria for ending embolization were stoppage of blood flow in the tumor blood vessel and complete disappearance of tumor staining under imaging.

### Efficacy, safety, and survival evaluation

Treatment response was assessed by enhanced computed tomography or magnetic resonance imaging (CT/MRI) in terms of the modified RECIST (mRECIST) assessment for HCC (Lencioni & Llovet, 2010). Evaluation data of treatment response at 1 month, 3 months, and 6 months after treatment were collected for study analysis. Meanwhile, patients’ Karnofsky performance status (KPS) scores assessed at 1 month, 3 months, and 6 months after treatment were also collected. For safety assessment, the adverse events and the main liver function indexes (before treatment, seven days after treatment, and 30 days after treatment) were collected and analyzed as well. As for survival evaluation, according to the surveillance and follow-up records, time to progression (TTP) and overall survival (OS) were summarized and analyzed in the study.

### Statistical analysis

We performed statistical analyses using SPSS (Social Package for Social Sciences) 20.0 software (IBM, Chicago, IL). Descriptive statistical analysis of the clinicopathological data was performed using mean values, standard deviations, numbers, and percentages. The *χ*^2^ test or Fisher’s exact test was used to analyze the unordered categorical data, and the Wilcoxon rank sum test was used to analyze the ordered categorical data. Student’s *t* test was used to determine the quantitative data. The Kaplan–Meier method was used to plot the survival curves. Log-rank (Mantel-Cox) test was applied for determining survival analysis. *p* Values <.05 were considered statistically significant.

## Results

### Clinical characteristics of HCC patients

No difference was found between the HepaSpheres group and CalliSpheres group regarding all the characteristics of HCC patients (Table 1). The mean age was 65.5 ± 8.7 years in HepaSpheres group and was 64.3 ± 9.0 years in CalliSpheres group (*p*= .431). The number of males was 42 (70.0%) in the HepaSpheres group and was 53 (79.1%) in the CalliSpheres group (*p*= .238). In addition, the numbers of patients with ECOG score of 0, 1, and 2 were 37 (61.7%), 20 (33.3%), and 3 (5.0%) in HepaSpheres group, then were 42 (62.7%), 21 (31.3%), as well as 4 (6.0%) in CalliSpheres group (*p*= .946). There were 34 (56.7%) and 26 (43.3%) patients in HepaSpheres group, and 40 (59.7%) and 27 (40.3%) patients in CalliSpheres group who had Child-Pugh stage A and B, respectively (*p*= .729). Besides, the mean value of tumor size was 7.9 ± 2.5 cm in the HepaSpheres group and was 7.5 ± 2.3 cm in the CalliSpheres group (*p*= .350). The numbers of patients in BCLC B stage and C stage were 27 (45.0%) and 33 (55.0%) in the HepaSpheres group, and were 32 (47.8%) and 35 (52.2%) in the CalliSpheres group (*p*= .755). The information of other characteristics is displayed in Table 1.

**Table 1.** Characteristics of HCC patients.

Items	HepaSpheres group (*N* = 60)	CalliSpheres group (*N* = 67)	*p* Value
Age (years), mean ± SD	65.5 ± 8.7	64.3 ± 9.0	.431
Gender, no. (%)			.238
Male	42 (70.0)	53 (79.1)	
Female	18 (30.0)	14 (20.9)	
History of liver disease, no. (%)			.994
Hepatitis B	48 (80.0)	53 (79.1)	
Hepatitis C	6 (10.0)	7 (10.4)	
ALD	3 (5.0)	3 (4.5)	
Others	3 (5.0)	4 (6.0)	
ECOG PS score, no. (%)			.946
0	37 (61.7)	42 (62.7)	
1	20 (33.3)	21 (31.3)	
2	3 (5.0)	4 (6.0)	
MELD score, mean ± SD	9.5 ± 2.2	9.3 ± 2.0	.592
Child-Pugh stage, no. (%)			.729
A	34 (56.7)	40 (59.7)	
B	26 (43.3)	27 (40.3)	
Tumor size (cm), mean ± SD	7.9 ± 2.5	7.5 ± 2.3	.350
Number of tumors, no. (%)			.821
≤3	37 (61.7)	40 (59.7)	
>3	23 (38.3)	27 (40.3)	
Vascular invasion, no. (%)			.767
Yes	13 (21.7)	16 (23.9)	
No	47 (78.3)	51 (76.1)	
Extrahepatic metastasis, no. (%)			.590
Yes	7 (11.7)	10 (14.9)	
No	53 (88.3)	57 (85.1)	
BCLC stage, no. (%)			.755
B	27 (45.0)	32 (47.8)	
C	33 (55.0)	35 (52.2)	
AFP level, no. (%)			.883
≤400 ng/mL	15 (25.0)	16 (23.9)	
>400 ng/mL	45 (75.0)	51 (76.1)	
Times of TACE treatment, mean ± SD	3.7 ± 3.3	3.4 ± 2.7	.574

HCC: hepatocellular carcinoma; SD: standard deviation; ALD: alcoholic liver disease; ECOG PS: Eastern Cooperative Oncology Group performance status; MELD: model for end-stage liver disease; BCLC: Barcelona Clinic Liver Cancer; AFP: alpha-fetoprotein; TACE: transarterial chemoembolization.

### Comparison of treatment response between the two groups

**Figure 1.**
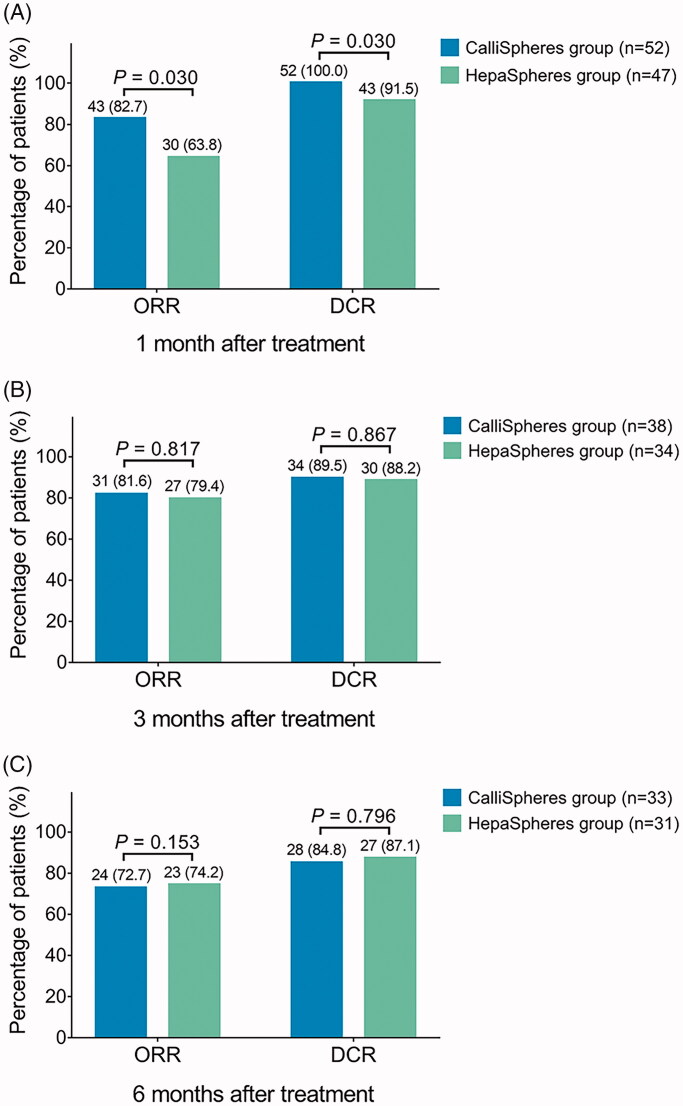
ORR and DCR in CalliSpheres group and HepaSpheres group. The comparison of ORR and DCR at 1 month (A), 3 months (B), and 6 months (C) after treatment between CalliSpheres group and HepaSpheres group. ORR: objective response rate; DCR: disease control rate.

The general treatment response was more favorable in the CalliSpheres group compared to HepaSpheres group, presenting as that the CR and PR were higher while SD was lower in CalliSpheres group compared to HepaSpheres group at 1 month after treatment, while there was no PD in CalliSpheres group (*p*= .026) (Table 2). However, the general treatment response at 3 months (*p*= .863) and 6 months (*p*= .853) were of no difference between the two groups. In terms of ORR and DCR, the ORR (*p*= .030) and DCR (*p*= .030) were both increased in CalliSpheres group compared to HepaSpheres group at 1 month after treatment (Figure 1(A)). However, the ORR (*p*= .817) and DCR (*p*= .867) at 3 months after treatment were similar between the two groups (Figure 1(B)); in addition, the ORR (*p*= .153) as well as DCR (*p*= .796) at 6 months were also of no difference between the two groups (Figure 1(C)).

**Table 2.** Treatment response.

Items	CR	PR	SD	PD	*p* Value
1 month after treatment					
CalliSpheres group (*n* = 52)	11 (21.2)	32 (61.5)	9 (17.3)	0 (0.0)	.026
HepaSpheres group (*n* = 47)	6 (12.8)	24 (51.1)	13 (27.8)	4 (8.5)	
3 months after treatment					
CalliSpheres group (*n* = 38)	8 (21.0)	23 (60.5)	3 (8.0)	4 (10.5)	.863
HepaSpheres group (*n* = 34)	7 (20.6)	20 (58.8)	3 (8.8)	4 (11.8)	
6 months after treatment					
CalliSpheres group (*n* = 33)	6 (18.2)	18 (54.5)	4 (12.1)	5 (15.2)	.853
HepaSpheres group (*n* = 31)	6 (19.4)	17 (54.8)	4 (12.9)	4 (12.9)	

CR: complete response; PR: partial response; SD: stable disease; PD: progressive disease.

### Comparison of performance status, liver function, and adverse events between the two groups

**Figure 2.**
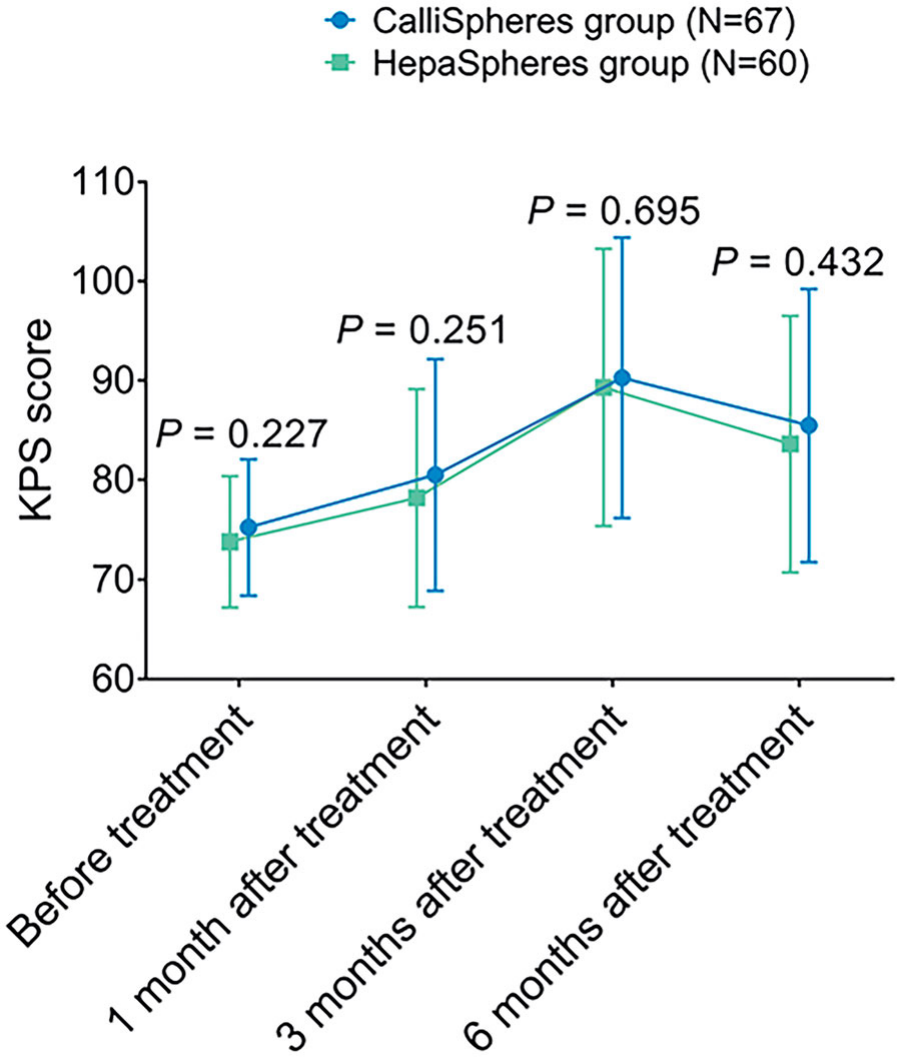
KPS score in CalliSpheres group and HepaSpheres group. The comparison of KPS score at 1 month, 3 months, and 6 months post treatment between CalliSpheres group and HepaSpheres group. KPS: Karnofsky performance status.

Moreover, the performance status by KPS was also compared between the CalliSpheres group and HepaSpheres group, which disclosed that the KPS score was similar between the two groups at 1 month (*p*= .251), 3 months (*p*= .695), and 6 months (*p*= .432) after treatment (Figure 2). The liver function index levels including ALT, AST, ALB, and TBIL at seven days and 30 days after treatment were similar between the CalliSpheres group and HepaSpheres group (all *p*> .05) (Table 3). In regard to the adverse events, which consisted of nausea/vomiting, pain, fever, myelosuppression, biloma, and abscess, were of no difference between the two groups (all *p*> .05) (Table 4). As for the adverse events in grade I–II, they were similar between the two groups as well (all *p*> .05), and so did the adverse events in grade III–IV (all *p*> .05).

**Table 3.** Liver function indexes.

Items	Before treatment	7 days after treatment	30 days after treatment
ALT (U/L), mean ± SD			
CalliSpheres group	42.7 ± 14.2	79.3 ± 23.5	39.3 ± 12.4
HepaSpheres group	41.9 ± 13.7	80.0 ± 24.2	40.1 ± 13.3
*p* Value	.748	.869	.726
AST (U/L), mean ± SD			
CalliSpheres group	49.0 ± 15.3	67.8 ± 21.9	53.4 ± 14.3
HepaSpheres group	48.9 ± 14.9	68.6 ± 20.9	54.0 ± 16.0
*p* Value	.970	.834	.824
ALB (g/L), mean ± SD			
CalliSpheres group	36.1 ± 9.9	34.2 ± 8.8	37.0 ± 11.4
HepaSpheres group	35.0 ± 8.9	34.8 ± 9.0	36.2 ± 10.6
*p* Value	.513	.705	.684
TBIL (mmol/L), mean ± SD			
CalliSpheres group	24.5 ± 10.1	27.0 ± 8.5	22.2 ± 6.9
HepaSpheres group	22.8 ± 7.3	24.6 ± 8.1	21.7 ± 7.1
*p* Value	.284	.107	.688

ALT: alanine aminotransferase; AST: aspartate aminotransferase; ALB: albumin; TBIL: total bilirubin.

**Table 4.** Adverse events.

Parameters	Total adverse events			Grade I–II			Grade III–IV		
	CalliSpheres group	HepaSpheres group	*p* Value	CalliSpheres group	HepaSpheres group	*p* Value	CalliSpheres group	HepaSpheres group	*p* Value
Nausea/vomiting, no. (%)	42 (62.7)	37 (61.7)	.906	39 (58.2)	35 (58.3)	.989	3 (4.5)	2 (3.3)	1.000
Pain, no. (%)	27 (40.3)	24 (40.0)	.973	25 (37.3)	22 (36.7)	.940	2 (3.0)	2 (3.3)	1.000
Fever, no. (%)	21 (31.3)	18 (30.0)	.870	19 (28.4)	17 (28.3)	.998	2 (3.0)	1 (1.7)	1.000
Myelosuppression, no. (%)	3 (4.5)	2 (3.3)	1.000	3 (4.5)	2 (3.3)	1.000	0 (0.0)	0 (0.0)	–
Biloma, no. (%)	1 (1.5)	1 (1.7)	1.000	1 (1.5)	1 (1.7)	1.000	0 (0.0)	0 (0.0)	–
Abscess, no. (%)	0 (0.0)	1 (1.7)	.472	0 (0.0)	1 (1.7)	.472	0 (0.0)	0 (0.0)	–

### Comparison of survival profile between the two groups

**Figure 3.**
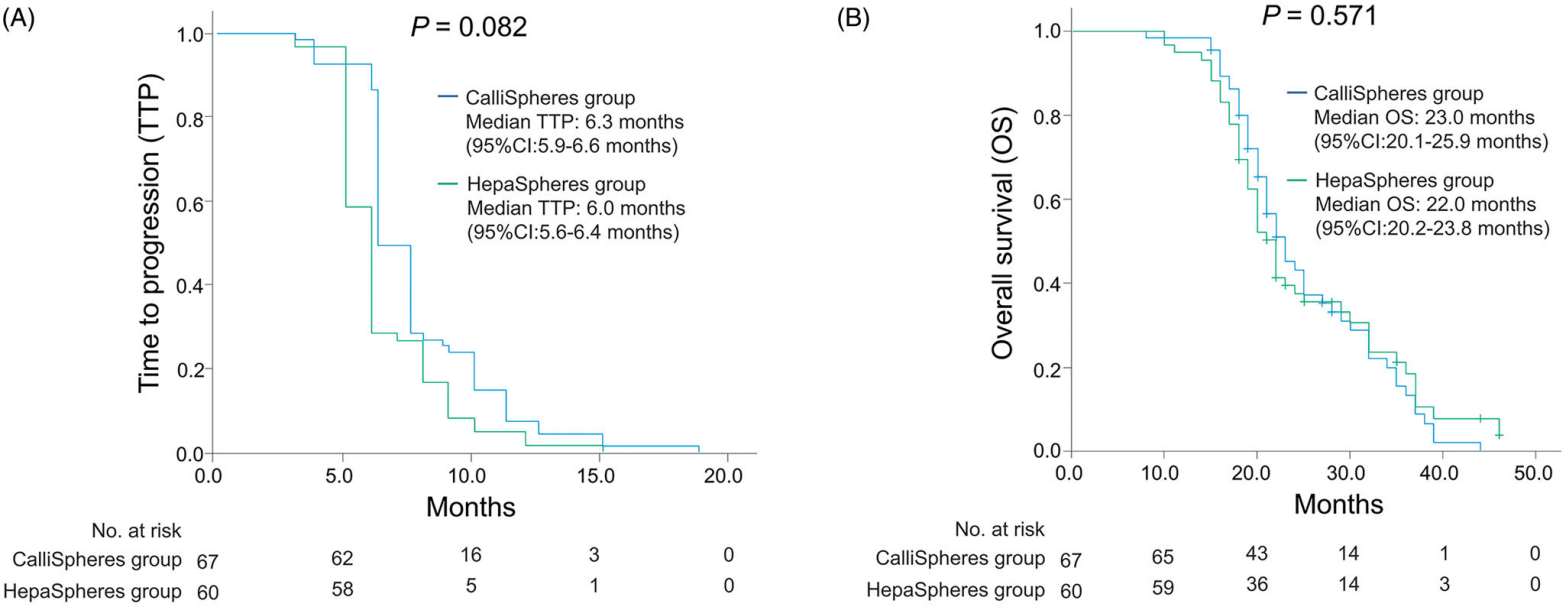
TTP and OS in CalliSpheres group and HepaSpheres group. The comparison of TTP (A) and OS (B) between CalliSpheres group and HepaSpheres group. TTP: time to progression; OS: overall survival; 95%CI: 95% confidence interval.

The TTP was similar between the CalliSpheres group and HepaSpheres group, and the median value of TTP was 6.3 months (95%CI: 5.9–6.6 months) in the CalliSpheres group, and was 6.0 months (95%CI: 5.6–6.4 months) in the HepaSpheres group (*p*= .082) (Figure 3(A)). As for OS, it was also of no difference between the two groups, and the median value of OS was 23.0 months (95%CI: 20.1–25.9 months) in the CalliSpheres group, and was 22.0 months (95%CI: 20.2–23.8 months) in the HepaSpheres group (*p*= .571) (Figure 3(B)). In addition, the numbers of patients at risk regarding TTP and OS were also shown at the bottom of Figure 3(A,B), respectively.

## Discussion

HCC is an aggressive and thus hard to treat solid caner, often presenting with advanced disease at the time of diagnosis that blocks many potential curative therapies (Grandhi et al., 2016; Chedid et al., 2017). DEB-TACE is effective and safe for HCC patients, although conventional TACE is still the mainstay of TACE treatments, the utilization of DEB-TACE presents with a growing trend in cancer patients. As for other embolization therapies, there is evidence elucidating that radioembolization and bland embolization are comparable regarding efficacy when compared to the chemoembolization in liver cancer patients; however, more efforts are needed to compare the efficacy and safety among different TACE treatments in liver cancer patients (Facciorusso et al., 2016, 2017). However, most authors emphasize the superiority or equal value of DEB-TACE compared to conventional TACE in treating HCC, very few authors investigate the impact of the microsphere category on efficacy and safety. For instance, there are many studies reporting the efficacy of DEB-TACE using HepaSpheres in HCC patients. A previous cohort study with 30 HCC patients treated by DEB-TACE using 50–100 μm HepaSpheres reveals that, at 1 month post treatment, the ORR is 63.3% and the DCR is 86.7% (Sun et al., 2017). Another prospective cohort study illustrates that in 18 HCC patients treated with DEB-TACE, the application of 50–100 μm HepaSpheres achieves an ORR of 53.3%, and the BCLC stage is correlated with treatment response rate (Zurstrassen et al., 2017). In regard to the CalliSpheres, a cohort study illuminates that in 50 middle stage HCC patients treated with DEB-TACE using CalliSpheres, CR, PR, SD, and PD are 35.4%, 29.4%, 17.6%, and 17.6%, respectively, in patients treated with 100–300 μm beads, and are 33.1%, 23.1%, 20.8%, as well as 23.0% respectively in patients treated with 300–500 μm beads (Wang et al., 2020). Another study consisting of a cohort of 90 HCC patients receiving DEB-TACE treatment using CalliSpheres reports that, the DCR at 3 months, 6 months, 1 year, and 2 years post treatment are 93.33%, 88.89%, 36.67%, and 12.22%, respectively; in addition, the 2-year survival rate is 45.56% (Cao et al., 2019).

In terms of the efficacy of DEB-TACE for patients with large HCC tumor, the studies are very rare, only few studies could be referred to. For instance, in a study of 81 elderly patients with advanced HCC with the largest tumor size of 5–10 cm, DEB-TACE using 300–500 μm CalliSpheres achieves a DCR of 75.8%, 42.4%, and 12.1% at 1, 3, and 6 months post treatment, respectively (Yang et al., 2020). Nevertheless, no effort has been done to explore the effect of different microsphere products on the efficacy and safety of DEB-TACE for treating patients with unresectable large HCC. In the present study, we found that the general treatment response of unresectable large HCC patients at 1 month after treatment was more favorable by CalliSpheres compared to HepaSpheres, but the treatment response at 3 months or 6 months after treatment was not. Additionally, the KPS scores at 1 month, 3 months, and 6 months after treatment were of no difference between the two groups. In terms of survival profile, there was no difference regarding TTP or OS between the two groups, either. For the increased treatment response rates by CalliSpheres compared to HepaSpheres at 1 month after treatment, we presumed that it could be caused by the following reasons. One of the possible reasons was that, the drug elution speed was lower when the microsphere diameter was greater, which indicated that the CalliSpheres (300–500 μm) used in our study was better in the slow-release effect of the chemotherapeutics compared to HepaSpheres (50–100 μm), and this might contribute to the more favorable efficacy by CalliSpheres at 1 month after treatment (Han et al., 2019; Chen et al., 2020, 2021). Another reason could be that, HepaSpheres is reported to present with fractures during the release of the drug, which could weaken the embolization effect in the vessel, thus caused a worse tumor necrosis effect (Jordan et al., 2010).

Regarding the safety profile, a previous study uncovers that in 15 patients (colorectal cancer liver metastasis and intrahepatic cholangiocarcinoma) treated with DEB-TACE using HepaSpheres, there are no severe adverse events during and post the treatment, with the most common adverse event of embolization syndrome (Poggi et al., 2008). Besides, another study reports that in unresectable HCC patients treated with DEB-TACE using HepaSpheres, the incidence of embolization syndrome, treatment related mortality and treatment related morbidity are 89%, 1.9%, and 9.4%, respectively (Kucukay et al., 2015). In regard to the safety profile of CalliSpheres, the rates of embolization syndrome, transient liver injury, liver abscess, ascites, myelosuppression, and granulocyte reduction are 62.5%, 46%, 4.1%, 13%, 4.1%, and 8.3%, respectively (Wu et al., 2018). In addition, a prospective cohort study elucidates that the incidences of liver function injury, pain, nausea, vomiting, and fever are 43.9%, 40.9%, 33.3%, 19.7%, and 56.1%, respectively, in 66 HCC patients treated with CalliSpheres DEB-TACE (Zhang et al., 2019). As for the safety of DEB-TACE in patients with large HCC, one study reveals that using 300–500 μm CalliSpheres in DEB-TACE to treat elderly patients with large HCC, no severe complication exists and the most common adverse event is embolization syndrome (Yang et al., 2020). In this study, we evaluated the liver function index levels at seven days and 30 days and adverse events after treatment, they were of no difference between the HepaSpheres group and CalliSpheres group. These indicated that CalliSpheres was equally tolerable compared to HepaSpheres in DEB-TACE for treatment of patients with large HCC. However, in the clinical setting, CalliSpheres is much more economical compared to HepaSpheres, which might indicate that patient could obtain comparable efficacy and safety but cost less if choosing CalliSpheres for DEB-TACE treatment (Kadam & Chuan, 2016; Zhou et al., 2018).

Another issue that should be discussed here was the limitation of our study. First, this was a retrospective observational study, which could cause some bias, such as the information bias. Second, the sample of 127 HCC patients was relatively small and this may interfere with our statistical power to some extent. Third, merely one size of each microsphere product (HepaSpheres and CalliSpheres) was evaluated in our study, thus, the impact of other microspheres sizes on efficacy and safety in unresectable large HCC patients, such as the 100–300 μm CalliSpheres, was still obscure.

In conclusion, CalliSpheres seems to be superior in short-term efficacy and equal in long-term efficacy as well as safety compared to HepaSpheres for DEB-TACE treatment in patients with unresectable large HCC.
